# A low-birth-weight risk assessment scale: development and validation through a questionnaire-based survey

**DOI:** 10.1186/s12913-019-3886-7

**Published:** 2019-01-18

**Authors:** Kazuko Sonoda, Yuko Matsunari, Syuji Takei

**Affiliations:** 1 Faculty of Fukuoka Medical Technology Teikyo Univaersity, 6-22, Misaki-machi, Omuta-shi, Fukuoka, 836-8505 Japan; 2 Department of Fundamental and Clinical Nursing School of Health Sciences, Faculty of Medicine, Kagoshima University, 8-35-1 Sakuragaoka, Kagoshima-shi, 890-8544 Japan; 3 Pediatrics, Developmental Medicine, Kagoshima University Graduate School of Medical and Dental Sciences, 8-35-1, Sakuragaoka, Kagoshima-shi, 890-8544 Japan

**Keywords:** Low birth weight, Risk assessment scale, Risk factor, Principal factor analysis, Promax rotation

## Abstract

**Background:**

Birth weight is continuously decreasing in Japan since food satiation has become a problem in recent years. The present study aimed to develop and examine the reliability and validity of a scale for the assessment of risk factors for low birth weight in infants born at term.

**Methods:**

A self-administered postal questionnaire survey comprising a low birth weight risk assessment scale was conducted on mothers with children of nursery school or kindergarten age. After item analysis (scale), factor structure was confirmed by an exploratory factor analysis using the main factor method promax rotation. The reliability of this scale was confirmed by Cronbach’s α coefficient and Item–Total correlation. The validity was confirmed by known-groups validity and internal validity.

**Results:**

The responses of 630 mothers (valid response rate, 18.5%) were analyzed. Factor analysis (principal factor analysis and promax rotation) obtained an optimal scale comprising 25 items in the following nine factors: “guidance at each checkup,” “adequate rest,” “support from husband,” “effects on the fetus,” “support from society,” “support from family,” “effects of minor troubles,” “good lifestyle habits,” and “fall risk and lifestyle changes.” The overall Cronbach’s α coefficient for the scale was 0.701. Known-groups validity examination revealed significant differences in scale scores of birth weight, birth history, and maternal smoking status.

**Conclusion:**

The scale demonstrated internal consistency, construct validity, and known-groups validity, indicating that it can be used as an indicator of low birth weight risk. In the future, this scale may be included in medical questionnaires as part of health guidance for pregnant women at a risk of delivering low birth weight children.

**Electronic supplementary material:**

The online version of this article (10.1186/s12913-019-3886-7) contains supplementary material, which is available to authorized users.

## Background

Three elements form the background to this study. First, the mean birth weight in Japan is continuously decreasing [1, 2], with Sagawa [1] reporting that the mean birth weight in 2011 was at least 200 g lower than that 30 years earlier. Birth weight is an indirect marker of intrauterine nutritional status, and fetal exposure to malnutrition or hypoxia leads to low birth weight [3]. Approximately half of the low-weight births are attributable to shorter gestational duration; however, an association with factors, such as maternal desire to be thin and psychosocial stress, has been indicated in the remaining cases [4]. Second, the frequency of low-weight births in Japan is increasing. The proportion of low birth weight infants in Japan decreased to 4.5% for males and 5.3% for females in 1976 and, thereafter, increased to 8.5% for males and 10.8% for females in 2010. The percentage of the increase in proportion of low birth weight children was 9.6%, after which it has remained stable [2].with 57% of the low birth weight infants born at term [5]. Third, according to the fetal origins of adult disease hypothesis [3], low birth weight is a risk factor for future lifestyle-related diseases. Therefore, it is important to clarify and work toward the mitigation of factors that contribute to low birth weight. Such efforts will promote the health of the future generations since low birth weight is not simply a perinatal care issue but a major health concern throughout the life.

The first step was to clarify the factors affecting birth weight. In a preliminary study, semi-structured interviews with 12 mothers of young children, including low birth weight infants, identified maternal lifestyle and psychological characteristics during pregnancy as potential causes of low birth weight [6]. We constructed original questionnaires based on the findings of this preliminary survey and previous studies [6] (Additional file 1) and used this questionnaire to develop an evaluation scale of maternal risk factors for low birth weight infants born at term.

## Methods

### Aim

The present study aimed to clarify the causal relationship between maternal daily lifestyle during pregnancy and low infant birth weight and to develop a scale for the assessment of maternity risk factors for low birth weight in infants born at term. A low birth weight risk assessment scale for use in prenatal checkups was developed, and its reliability and validity were examined.

### Subjects

Subjects were mothers with the youngest child below school age (≤6 years).

### Survey methods

This was a questionnaire-based survey. A total of 3400 questionnaires were distributed to 42 nursery schools and kindergartens in A Prefecture (including those in remote islands) between February and March 2015. After requesting and obtaining consent from the heads of the Nursery School Association and Private Kindergarten Association Recommended Facilities in A Prefecture, the nursery schools and kindergartens distributed questionnaire packages (containing a description of the survey, a self-administered questionnaire, and a return envelope) to parents and guardians. Postal return of the completed questionnaire to the researchers using the return envelope was considered as the final consent for participation.

### Survey content

Questions regarding maternal characteristics included maternal age, height, pre-pregnancy weight, age at the time of delivery, weight gain during pregnancy, participation in classes for mothers, employment status, and the number of children in the family. Questions for pre-school children included age, sex, birth weight, and gestational age at birth. In addition, a draft scale prepared by the below mentioned method was included(Additional file 1).

The preliminary study identified variables of maternal pre-pregnancy malnutrition, low weight gain, and smoking during pregnancy as factors contributing to low birth weight [6]. These findings together with causes of low birth weight identified in previous research [4, 7, 8] were reflected in the scale items. Following basic procedures for questionnaire design, the scale was developed on the basis of the preliminary survey results and previous research [3, 7, 8], and the repeated review of the scale was conducted until an agreement was reached among the joint authors and then with public health nurses with hospital-based clinical experience. Efforts were then made to ensure the accuracy and validity of the scale, seeking repeat opinions from a researcher/midwife instructor of maternal and child health nursing. Through these steps, the scale was organized into 33 items.

A four-point rating scale with the neutral mid-point eliminated was used to accurately confirm respondents’ opinions (1, strongly disagree; 2, slightly disagree; 3, slightly agree; and 4, strongly agree). Responses were scored from 1 to 4 points to attain a score for each item. The magnitude of the score demonstrated the degree of relevance to risk factors for low birth weight.

### Analysis

In order to assess subject characteristics, descriptive statistics were calculated for all variables, and characteristics were then selected for inclusion in the scale on the basis of the item analysis criteria. According to their employment status during pregnancy, women were classified as “non-working” if they continued as full-time homemakers during pregnancy or “working” if they were regular or non-regular employees or were self-employed during pregnancy. Statistical package SPSS 20.0 was used for the analysis with significance set at *p* < 0.05.

The scale was developed on the basis of item analysis, and scale reliability and validity were then examined. The inclusion of each item was determined as follows: (i) the presence of floor or ceiling effects was confirmed on the basis of mean values and standard deviations (average value + standard deviation was calculated for the ceiling effect, and when this value exceeded the maximum value of 4 points, it was considered that there was a ceiling effect. When the average value − standard deviation was smaller than the minimum value of 1 point, it was considered that there was a floor effect); (ii) Good–Poor analysis [G–P analysis; t test was performed to assess the difference between the mean values of the upper and lower thirds of the total scores (upper group and lower group, respectively) for each item, and items with a significant difference were retained] was performed; and (iii) inter-item correlation matrix was obtained.

The reliability of the scale was determined on the basis of the Cronbach’s α coefficient and correlation analysis between the item and scale [Item–Total correlation] to investigate item inclusion. The validity of the scale was examined as follows: (i) content validity of the scale was investigated; (ii) construct validity was investigated using exploratory factor analysis; and (iii) known-groups validity was investigated. Concurrent validity could not be investigated as no adequate scale exists for investigating criterion validity; therefore, known-groups validity was tested. The following three variables according to their known-groups validity were classified and subjected to comparative analysis of the mean scale total scores: (i) birth weights classified as “< 2500 g” or “≥2500 g” (t-test); (ii) birth histories classified as “primipara” or “multipara” (t-test); and (iii) maternal smoking status classified into the following five groups: “no history of smoking,” “stopped smoking before this pregnancy,” “permanently stopped smoking during this pregnancy,” “temporarily stopped smoking during this pregnancy,” and “continued smoking throughout this pregnancy” [one-way analysis of variance (ANOVA) and Tukey–Kramer honestly significant difference (HSD) test].

### Ethical considerations

The study aims and outline, subject rights (voluntary nature of participation and cooperation and right to refusal to participate), protection of privacy through questionnaire anonymity, use of ID numbers instead of names, protection of personal information, data confidentiality, restriction of data use to the present study, destruction of questionnaires immediately after finishing the present study, and the fact that returning a completed questionnaire would be considered as consent for participation were explained to all subjects.

## Results

### Status of questionnaire collection and subject characteristics

Out of the 3400 questionnaires distributed, responses were received from 671 mothers (collection rate, 19.7%). The aim of this study was to construct a scale to assess pregnant women who may deliver a low birth weight baby because of their lifestyles, which may affect the development of the fetus lurking in their daily life. Responses from mothers of twins or sick babies, which are conditions that are considered to be associated with low birth weight, were excluded from the analysis. In total, the responses from 630 mothers of babies (valid response rate, 18.5%) and 916 mothers of pre-school children were analyzed.

Maternal characteristics were as follows: mean age, 35.7 [standard deviation (SD)], 4.9; range, 23–51) years; mean number of children in the family, 2.2 (SD, 0.9; range, 1–8); mean age at time of delivery, 31.2 (SD, 4.6, range, 20–50) years; and employment status during pregnancy, 397 non-working (43.3%) and 519 working (56.7%). Child characteristics were as follows: mean age, 3.6 (SD, 1.8; range, 0–6) years; sex, 463 boys (53.3%) and 406 girls (46.7%) (Table 1).

**Table 1:** Subject characteristics (mothers, *n* = 630; the youngest children, *n* = 916)

Items	*n* (%)	Mean (SD)	Range
Maternal age (years; *n* = 629*)		35.7 (4.9)	23–51
Number of children in the family		2.2 (0.9)	1–8
Child age (years; *n* = 914*)		3.6 (1.8)	0–6
Child sex (*n* = 869*)			
Boys	463 (53.3)		
Girls	406 (46.7)		
Child weight at birth (g)		3005.7 (423.5)	722–4320
< 2500	89 (9.7)	2201.7 (333.8)	722–2498
≥2500	827 (90.3)	3092.2 (331.1)	2500–4320
Gestational age at birth (weeks; *n* = 884)	38.7 (1.7)		25–42
Maternal age at birth (years; *n* = 909)	31.2 (4.6)		20–50
Birth history			
Primipara	347 (37.9)		
Multipara	569 (62.1)		
Maternal weight gain during pregnancy (kg; *n* = 895)	10.1 (3.7)		−4.0–27.0
Maternal employment status during pregnancy			
Non-working	397 (43.3)		
Working	519 (56.7)		

*Excluded undescribed data

### Developing the scale

The response rate for all 33 items in the scale was ≥99.5%. The scores for each item ranged from 1 to 4, with mean scores of 1.1–3.8 (SD, 0.5–1.2) (Table 2). The confirmation of the data distribution revealed a floor or ceiling effelatict for items 2, 5–7, 10, 12, 22–24, 27, 30, and 32. However, these items were considered necessary to investigate factor structure and were included in the factor analysis. On G–P analysis, significant differences were observed between the upper and lower groups for all items (Table 2). Therefore, all items were judged to adequately correspond with the total scores and were included in the scale.

**Table 2:** Standard distributions for the low birth weight risk assessment scale items (*n* = 916)

Items	Response rate *n* = 916%	Strongly Disagree *n* (%)	Slightly Disagree *n* (%)	Slightly Agree *n* (%)	Strongly Agree *n* (%)	Mean ± SD
(1) I had severe morning sickness	99.9	149 (16.3)	251 (27.4)	298 (32.5)	217 (23.7)	2.6 ± 1.0
(2) At one point, I believed that severe morning sickness must be hereditary	99.2	379 (41.4)	357 (39.0)	119 (13.0)	54 (5.9)	1.8 ± 0.9
(3) I experienced unpleasant symptoms such as headaches	100.0	263 (28.7)	291 (31.8)	249 (27.2)	113 (12.3)	2.2 ± 1.0
(4) During pregnancy checkups, I was given advice on my health status^a^	99.8	294 (32.1)	272 (29.7)	243 (26.5)	105 (11.5)	2.2 ± 1.0
(5) I consumed alcohol^a^	100.0	813 (90.7)	56 (6.1)	20 (2.2)	9 (1.0)	1.1 ± 0.5
(6) I thought about the effects of alcohol consumption on the fetus	99.3	203 (22.2)	45 (4.9)	118 (12.9)	544 (59.4)	3.1 ± 1.2
(7) I thought about the effects of smoking on the fetus	99.8	143 (15.6)	42 (4.6)	95 (10.4)	634 (69.2)	3.3 ± 1.1
(8) I was always concerned about weight gain	100.0	66 (7.2)	186 (20.3)	311 (34.0)	353 (38.5)	3.0 ± 0.9
(9) During pregnancy checkups, I was often given advice about my weight gain by medical staff	100.0	284 (31.0)	273 (29.8)	234 (25.5)	125 (13.6)	2.2 ± 1.0
(10) I did not like going to checkups because the medical staff frequently advised me about my weight gain during previous pregnancy checkups	99.6	504 (55.0)	243 (26.5)	103 (11.2)	62 (6.8)	1.7 ± 0.9
(11) I restricted my food intake as I was concerned about gaining weight	99.8	316 (34.5)	302 (33.0)	211 (23.0)	85 (9.3)	2.1 ± 1.0
(12) I restricted my salt intake as I was concerned about my blood pressure	99.9	443 (48.4)	269 (29.4)	147 (16.0)	56 (6.1)	1.8 ± 0.9
(13) I often experienced abdominal bloating^a^	99.9	192 (21.0)	282 (30.8)	237 (25.9)	204 (22.3)	2.5 ± 1.1
(14) I was able to lead an orderly lifestyle	100.0	47 (5.1)	223 (24.3)	456 (49.8)	190 (20.7)	2.9 ± 0.8
(15) I tried to eat well-balanced meals	100.0	38 (4.1)	193 (21.1)	503 (54.9)	182 (19.9)	2.9 ± 0.8
(16) I exercised adequately	100.0	100 (10.9)	376 (41.0)	334 (36.5)	106 (11.6)	2.5 ± 0.8
(17) I slept well	100.0	65 (7.1)	278 (30.3)	370 (40.4)	203 (22.2)	2.8 ± 0.9
(18) When I woke from my sleep, I felt refreshed	100.0	78 (8.5)	343 (37.4)	378 (41.3)	117 (12.8)	2.6 ± 0.8
(19) I took holidays	100.0	58 (6.3)	231 (25.2)	371 (40.5)	256 (27.9)	2.9 ± 0.9
(20) I took adequate rest	99.9	51 (5.6)	219 (23.9)	401 (43.8)	244 (26.6)	2.9 ± 0.9
(21) At times I felt stressed^a^	99.8	84 (9.2)	251 (27.4)	393 (42.9)	186 (20.3)	2.8 ± 0.9
(22) I switched to shoes with low heels	99.7	29 (3.2)	28 (3.1)	101 (11.0)	755 (82.4)	3.7 ± 0.7
(23) I was careful not to fall	100.0	13 (1.4)	26 (2.8)	135 (14.7)	742 (81.0)	3.8 ± 0.6
(24) I refrained from driving a car^a^	99.7	640 (69.9)	137 (15.0)	62 (6.8)	74 (8.1)	1.5 ± 0.9
(25) There were some changes in my lifestyle compared to my pre-pregnancy days^a^	99.7	110 (12.0)	285 (31.1)	283 (30.9)	235 (25.7)	2.7 ± 1.0
(26) My husband thoroughly supported me	99.9	71 (7.8)	220 (24.0)	309 (33.7)	315 (34.4)	3.0 ± 0.9
(27) My parents (including in-laws) thoroughly supported me	100.0	55 (6.0)	125 (13.6)	286 (31.2)	450 (49.1)	3.2 ± 0.9
(28) People other than my husband and parents (including in-laws) thoroughly supported me	99.9	199 (21.7)	247 (27.0)	290 (31.7)	179 (19.5)	2.5 ± 1.0
(29) I am satisfied with my husband’s support	99.8	112 (12.2)	216 (23.6)	293 (32.0)	293 (32.0)	2.8 ± 1.0
(30) I am satisfied with my parents’ (including in-laws) support	100.0	42 (4.6)	121 (13.2)	301 (32.9)	452 (49.3)	3.3 ± 0.9
(31) I am satisfied with the support that I received from others besides my husband and parents (including in-laws)	99.8	149 (16.3)	186 (20.3)	298 (32.5)	281 (30.7)	2.8 ± 1.1
(32) I took advantage of the Maternity Mark (a symbol for expectant mothers) and experienced the effectiveness of the symbol in public settings^a^	99.5	443 (48.4)	278 (30.3)	132 (14.4)	58 (6.3)	1.8 ± 0.9
(33) I thought that the saying, “deliver them small and raise them big”, held true for me and my child(ren)^a^	99.7	191 (20.9)	294 (32.1)	248 (27.1)	180 (19.7)	2.5 ± 1.0

Means were calculated by scoring responses as follows: “strongly disagree” = 1 point; “slightly disagree” = 2 points; “slightly agree” = 3 points, and “strongly agree” = 4 points

^a^excluded items; underlined numbers indicate items with floor or ceiling effects; *SD* standard deviation

**Fig. 1:**
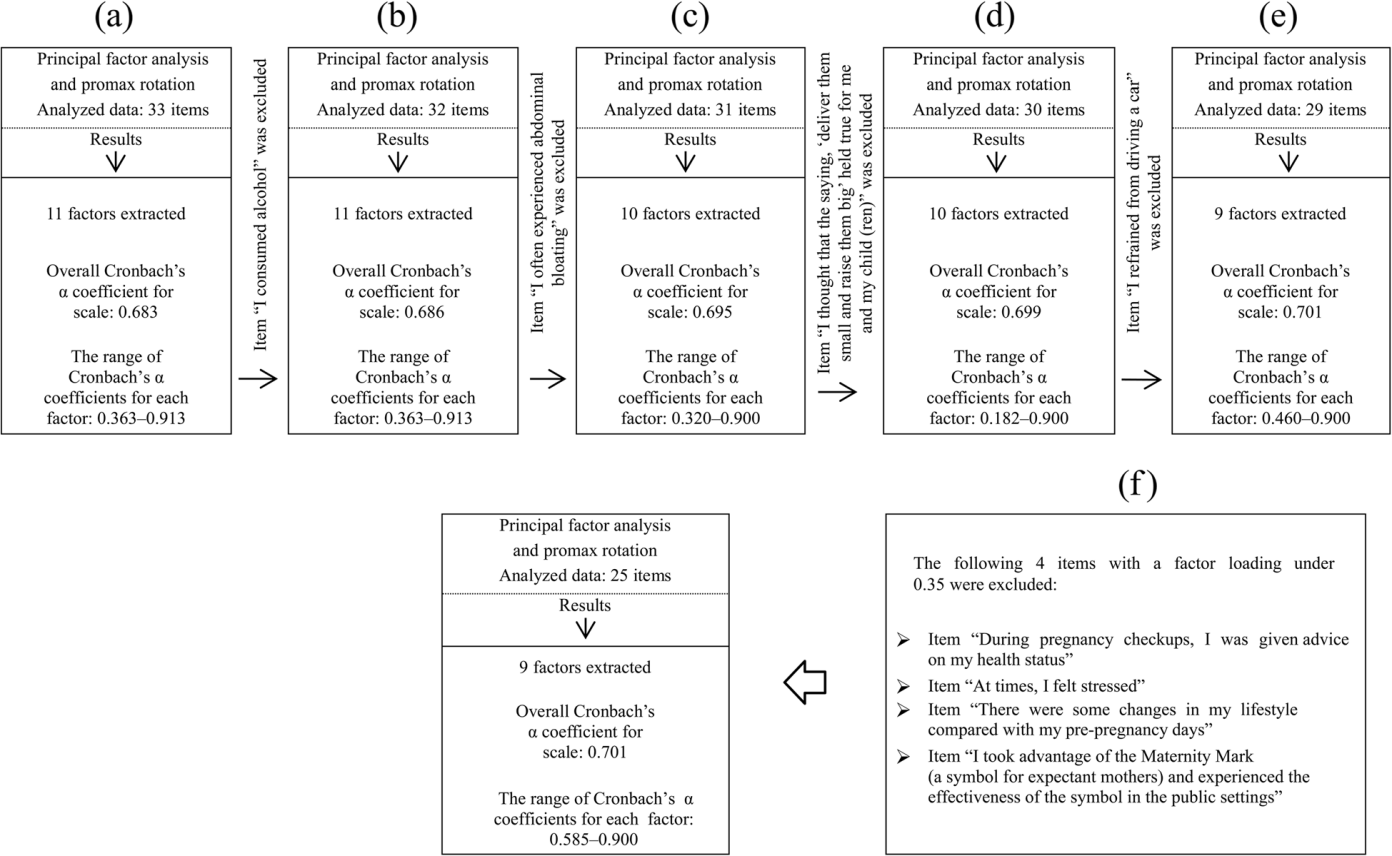
Factor analysis. **a** A total of 11 factors were extracted with factor analysis using principal factor analysis and promax rotation conducted on 33 items. The 11th factor had a low Cronbach’s α coefficient of 0.363. **b** Item 5, “I consumed alcohol,” which had the lowest coefficient value in the inter-item correlation matrix, was excluded, and factor analysis was repeated; however, Cronbach’s α coefficient remained low. **c** Item 13, “I often experienced abdominal bloating,” was excluded, and factor analysis was repeated on 31 items. 10 factors were extracted; however, the 10th factor had a low Cronbach’s α coefficient. **d** Item 33, “I thought that the saying, ‘deliver them small and raise them big’ held true for me and my child(ren),” was excluded, and factor analysis was repeated on 30 items; however, Cronbach’s α coefficient for the 10th factor remained low. **e** Item 24, “I refrained from driving,” was excluded, and factor analysis was repeated for 29 items. Finally, 9 factors were extracted, and the lowest Cronbach’s α coefficient increased to 0.460. **f** Four items (items 4, 21, 25 and 32) with factor loading under 0.35 were excluded, and factor analysis was repeated, finally extracting nine factors. The lowest Cronbach’s α coefficient for these nine factors was 0.585, and the overall Cronbach’s α coefficient for entire scale was 0.701

Eleven factors were extracted on factor analysis using principal factor analysis and promax rotation of the responses for all 33 items. An eigenvalue cutoff of ≥1 was used to determine the number of factors to retain. Cronbach’s α coefficient for the 11th factor was low (0.363). Therefore, the item “I consumed alcohol”, which had the greatest number of low coefficient values in the inter-item correlation matrix, was excluded, and factor analysis was repeated for the remaining 32 items. However, Cronbach’s α coefficient for the 11th factor remained low; therefore, the factor analysis process was repeated until Cronbach’s α coefficient increased and the results stabilized. The following items were eliminated from subsequent analyses: “I consumed alcohol,” “I often experienced abdominal bloating,” “I thought that the saying, ‘deliver them small and raise them big,’ held true for me and my child(ren),” and “I refrained from driving a car.” Factor analysis was conducted on the remaining 29 items, and the lowest Cronbach’s α coefficient increased to 0.460. At this point, nine factors were extracted. Factor analysis was conducted again excluding four items with a factor loading of under 0.35 (items 4, 21, 25, and 32). As a result, an optimal scale was obtained, which was interpreted without contradictions in the meanings of the items. The final scale comprised 25 items in 9 factors. The details and results of this analysis are shown in Fig. 1.

Kaiser–Meyer–Olkin test with the sampling adequacy of 0.640 and the screen plot with changes in factor eigenvalues indicated that 9 factors were appropriate. The cumulative contribution ratio of the sum of squared load amount after extraction was 57.0%. Therefore, items with factor loading ≥0.35 were included in the scale, and subsequent examinations of reliability and validity were conducted for 25 items in 9 factors. The factor pattern and factor correlation matrix are shown in Table 3.

**Table 3:** Results of factor analysis for low birth weight risk assessment scale (25 items)

Items	Reliability (Cronbach’s α)	Factor loadings									n	Mean	SD	Range
		1	2	3	4	5	6	7	8	9				
1. Guidance at each checkup	0.775										909	10.8	3.5	5–20
I restricted my food intake as I was concerned about gaining weight		0.727												
During pregnancy checkups, I was often given advice about my weight gain by medical staff		0.723												
I did not like going to checkups because the medical staff frequently advised me about my weight gain during previous pregnancy checkups		0.716												
I was always concerned about weight gain		0.630												
I restricted my food intake as I was concerned about gaining weight		0.425												
2. Adequate rest	0.778										915	11.2	2.7	4–16
I took adequate rest			0.923											
I took holidays			0.898											
I slept well			0.428											
When I woke from my sleep, I felt refreshed			0.399											
3. Support from husband	0.900										914	5.8	1.9	2–8
I am satisfied with my husband’s support				0.901										
My husband thoroughly supported me				0.898										
4. Effects on fetus	0.895										910	6.4	2.3	2–8
I thought about the effects of alcohol consumption on the fetus					0.910									
I thought about the effects of smoking on the fetus					0.899									
5. Support from society	0.861										913	5.3	2.0	2–8
People other than my husband and parents (including in-laws) thoroughly supported me						0.904								
I am satisfied with the support that I received from others besides my husband and parents (including in-laws)						0.809								
6. Support from family	0.853										916	6.5	1.7	2–8
I am satisfied with my parents’ (including in-laws) support							0.901							
My parents (including in-laws) thoroughly supported me							0.798							
7. Effects of minor troubles	0.648										908	6.7	2.2	3–12
I had severe morning sickness								0.818						
At one point, I believed that severe morning sickness must be hereditary								0.647						
I experienced unpleasant symptoms such as headaches								0.416						
8. Good lifestyle habits	0.585										916	8.3	1.8	3–12
I tried to eat well-balanced meals									0.687					
I was able to lead a regular lifestyle									0.520					
I exercised adequately									0.415					
9. Fall risk and lifestyle changes	0.697										913	7.5	1.1	2–8
I was careful not to fall										0.795				
I switched to shoes with low heels										0.676				
Scale total scores											888	68.5	8.1	32–93
Inter-factor correlation	1	1.00												
	2	−0.107	1.00											
	3	−0.005	0.191	1.00										
	4	0.069	− 0.007	−0.034	1.00									
	5	−0.018	0.132	0.342	0.016	1.00								
	6	0.009	0.186	0.395	0.026	0.417	1.00							
	7	0.147	−0.084	−0.018	0.136	−0.015	0.056	1.00						
	8	0.045	0.336	0.135	0.158	0.071	0.033	−0.117	1.00					
	9	0.135	0.051	0.084	0.234	0.080	0.135	0.053	0.131					

Overall Cronbach’s α coefficient, 0.701; cumulative contribution rate of nine factors extracted by principal factor analysis and promax rotation, 57.0%; SD, standard deviation

The 9 factors were labeled to reflect the related content assessment as follows. (i) guidance at each checkup: maternal feelings at the time of guidance (five items related to guidance from doctors and nurses regarding prenatal checkup findings); (ii) adequate rest: the state of maternal rest during pregnancy (four related items, such as maternal rest, holidays, and sleep); (iii) support from husband: support provided by the husband and related maternal satisfaction (two items); (iv) effects on the fetus: effects of alcohol consumption and tobacco on the fetus (two items); (v) support from society: social support other than that provided by the husband and family and related maternal satisfaction (two items); (vi) support from family: support provided by parents (including in-laws) and related maternal satisfaction (two items); (vii) effects of minor troubles: the extent of and related maternal feelings regarding minor troubles, such as morning sickness (three items); (viii) good lifestyle habits: good lifestyle habits during pregnancy (three items); and (ix) fall risk and lifestyle changes: concern regarding falling during pregnancy and associated lifestyle changes (two items).

### Association between factors of the scale

The strongest positive correlation in the factor correlation matrix was between “social support” and “family support” (0.417), followed by among “support from husband”, “support from society”, and “support from family,” between “adequate rest” and “good lifestyle habits” (0.336–0.395), and between “effects on the fetus” and “fall risk and lifestyle habits” (0.234).

These findings show a structure comprising two groups, one related to daily lifestyle during pregnancy, including “adequate rest,” “good lifestyle habits,” “effects on the fetus,” “effects of minor trouble,” “guidance at each checkup,” and “fall risk and lifestyle changes” and one related to support including “support from husband,” “support from family,” and “support from society.”

### Scale and item reliability

The mean scale total score was 68.5 (SD, 8.1; range, 32–93), and the normal probability–probability plot showed a normal distribution. The scale score was 25–100 points. Regarding the overall reliability of the scale, Cronbach’s α coefficient for each item ranged from 0.09–0.585 and that for the overall scale was 0.701 (Table 3). A positive Item–Total correlation was also observed.

### Scale and item validity

After confirming content validity among the authors, aspects of the scale, such as standard language among items and concept equivalence, were repeatedly evaluated by two authors, and two expert researchers in the field of maternal and child nursing summarized the items while maintaining their meaning to form the scale.

Exploratory factor analysis using principal factor analysis and promax rotation was performed to confirm the structure of the assessment indicators. Regarding birth weight, the mean subscale total scores of the ≥2500 g group were significantly higher than those of the < 2500 g group (t = − 3.153, *p* = 0.002). Regarding birth history, the mean total scores of primipara were significantly higher than those of multipara (t = 4.317, *p* < 0.00). One-way ANOVA revealed a significant difference in scores for maternal smoking status, whereas multiple comparison (Tukey–Kramer HSD test) demonstrated that the “no history of smoking” group had significantly higher mean total scores than the “continued smoking throughout this pregnancy” group (*p* < 0.00). The “stopped smoking before this pregnancy” group had significantly higher mean total scores than the “continued smoking throughout this pregnancy” group (*p* < 0.00), while the “permanently stopped smoking during this pregnancy” group also had significantly higher mean total scores than the “continued smoking throughout this pregnancy” group (*p* < 0.00) (Table 4).

**Table 4:** Relationship between total scores and birth weight, birth history, and maternal smoking status during pregnancy

Items	Group					t	F	*p*	Tukey–Kramer HSD test (*p* value)
Birth weight (g)	< 2500	≥2500 g							
Mean	65.9	68.7				−3.153		0.002	
(SD)	(8.8)	(8.0)							
*n*	86	802							
Birth history	Primipara	Multipara							
Mean	69.7	67.7				3.534		*p* < 0.0001	
(SD)	(8.6)	(7.7)							
*n*	334	554							
Maternal smoking status	No history of smoking	Stopped smoking before this pregnancy	Permanently stopped smoking during this pregnancy	Temporarily stopped smoking during this pregnancy	Continued smoking throughout this pregnancy				No history of smoking > Continued smoking throughout this pregnancy (*p* < 0.0001) Stopped smoking before this pregnancy > Continued smoking throughout this pregnancy (*p* < 0.0001) Permanently stopped smoking during this pregnancy > Continued smoking throughout this pregnancy (*p* < 0.0001)
Mean	68.9	69.6	69.2	65.8	59.8		7.894	*p* < 0.0001	
(SD)	(7.7)	(8.4)	(7.8)	(6.9)	(8.5)				
*n*	579	130	53	17	21				

t test, one-way analysis of variance; *p* < 0.05. *HSD* honestly significant difference, *SD* standard deviation

## Discussion

The present study investigated the reliability and validity of a low birth weight risk scale for the assessment of maternity risk factors for low birth weight in infants born at term. In this section, we discuss scale reliability and validity, characteristics of the extracted construct, as well as scale application and future issues.

### Data relevance

Regarding subject characteristics, the number of children in a family in the present study was slightly higher than that reported in the birth rates by maternal age group, number of live births by birth order, and the sex distribution of infants in the population for 2013 [2], while maternal age at the time of birth and the sex of the child were similar to the reported values. Valid responses were obtained from 630 mothers and 916 young children, and the data were normally distributed; thus, the data volume was sufficient to examine reliability and validity.

### Theoretical structure of the scale

#### Item analysis

Although item analysis revealed floor or ceiling effects for 12 items, these items were retained in the scale. Yoshida et al. [4] have reported that approximately half of low-weight births can be explained by shorter gestational duration. They found the remaining cases to be associated with maternal desire to be thin, weight gain, smoking, and psychosocial stress during pregnancy, while they found no correlation with putative causes, such as maternal age and birth order. The scale items used in the present study reflected the results of an interview survey with mothers of young children and included weight gain during pregnancy, maternal feelings regarding medical guidance concerning weight, and maternal values regarding pregnancy and childbirth. Therefore, although the ceiling effect is a negative aspect of this low birth weight risk assessment scale, the scores for these items can be applied as indicators of maternal health and were thus subjected to analysis without alteration.

#### Reliability (internal consistency)

The four items with the lowest correlation coefficients on item analysis and in the inter-item correlation matrix were eliminated from the scale. Furthermore, after factor analysis of 29 items, the 4 items with a factor loading under 0.35 after rotation were also excluded, leaving 25 items remaining. Although the reliability coefficients (Cronbach’s α) for the 8th factor were low, they were within the acceptable range for the present study, which combined 25 items into one questionnaire. Thus, internal consistency was ensured, and reliability of the scale was confirmed.

#### Validity

Content validity of the scale was ensured by repeated confirmation among the authors during the scale development. The results of exploratory factor analysis to investigate construct validity showed a nine-factor structure for the low birth weight risk assessment scale. These nine factors cover all of the important items capable of assessing risk factors for low birth weight, indicating construct validity of the present scale. The 1st factor is an indicator of the extent of self-monitoring during pregnancy related to indications and guidance received during prenatal checkups. Prenatal checkups have important implications not only for the identification and subsequent management of abnormal cases but also for the provision of guidance regarding maternal lifestyle improvements in uneventful pregnancies. Maternal build, such as being underweight or obese, affects fetal development [4]. Prenatal checkup guidance is provided according to the recommended values for weight gain during pregnancy based on pre-pregnancy body mass index established by the Japan Society of Obstetrics and Gynecology [9].

The 2nd factor is an indicator of the degree of mental and physical rest obtained during pregnancy. Changes in hormonal balance together with the increasing size of the abdomen with the growth of the fetus exert a psychological effect, and the occurrence of minor troubles also affects daily life and employment during pregnancy. Therefore, adequate rest and good quality sleep enable recovery from pregnancy fatigue, minimize physical and mental effects, and are essential for promoting and maintaining self-care ability.

The 3rd, 5th, and 6th factors are indicators of the degree of support from the husband, family, and society and maternal satisfaction with such support. The presence of support can relieve maternal anxiety or emotional changes experienced during pregnancy [10]. Husbands and mothers are the most important supporters for primipara, playing major roles in approval (appraisal), empathy (emotional), and direct assistance support [10]; therefore, the 3rd and 6th factors are crucial.

The 4th factor is an indicator of effects on the fetus. Alcohol consumption during any stage of pregnancy is believed to affect the fetus, potentially resulting in miscarriage, deformity, brain damage, intrauterine growth retardation, or low birth weight [11]. Meanwhile, smoking during pregnancy has been shown to increase the risk of outcomes, such as premature birth, miscarriage, and abnormal fetal development. Therefore, initiatives are required to actively support the cessation of smoking from the perspective of avoiding fetal effects.

The 7th factor is an indicator of the extent of minor troubles. Morning sickness during the first trimester of pregnancy is a common cause of psychological conflict and, if severe, can cause difficulties while eating and, in some cases, severe dehydration or malnutrition. In addition to causing difficulties for the mother, this has effects on the fetus; therefore, related items are important in terms of effects on birth weight.

The 8th factor is an indicator of the extent to which positive habits are incorporated into the daily lifestyle. Being underweight before pregnancy and insufficient weight gain during pregnancy lead to poor fetal nutritional status, which can cause low birth weight [12]. Therefore, particular care is required during pregnancy to eat a well-balanced diet and to lead a regular lifestyle. In addition, exercise during pregnancy brings a sense of invigoration, and maintaining a positive emotional state improves general malaise.

Finally, the 9th factor is an indicator of maternal risk of falls. Falls during pregnancy are associated with the risk of miscarriage and premature birth; therefore, great care must be taken to avoid falling. Women in Japan tend to switch to lower heels during pregnancy [6].

#### Known-groups validity

No existing scale could be found for investigating concurrent validity; therefore, known-groups validity was verified. A significant difference was observed in the mean total subscale scores on inter-group comparison regarding birth weight, birth history, and maternal smoking status. The present findings are consistent with those of previous studies showing that infant birth weight tends to be lower among primipara compared with that in multipara [13] and that the risk of low birth weight for gestational age due to intrauterine growth retardation is 2.08 times higher with maternal smoking during pregnancy [14].

These findings demonstrate the validity of this scale comprising 25 items in 9 factors as a scale capable of assessing maternity risk factors for low birth weight in infants born at term.

### Applications and future issues

The reliability and validity of this maternity low birth weight risk assessment scale were verified; thus, it can be used for the screening of high-risk pregnancies during health guidance. This scale would be particularly useful for identifying high-risk pregnancies during obtaining the history of mothers in prenatal checkups or during consultations with pregnant employees in the labor management section in their company regardless of the ability of the consultant.

There are some limitations in the present study. This was a cohort study regarding the daily lifestyles of pregnant women. The survey method may have introduced recall bias as responses were obtained from women with children of nursery school or kindergarten age looking back at and recalling their pregnancy lifestyles. Ideally, the survey should have been conducted either during pregnancy or soon after birth. However, the present study used a systematic oral history method focusing on mothers with children of nursery school and kindergarten age who were able to reflect and recall their lifestyle and sense of values during pregnancy [15]. The scale also included items with floor or ceiling effects, and there were factors with low Cronbach’s α coefficients. Therefore, caution should be exercised when interpreting the results. Also, this survey was conducted in a single prefecture, and conducting further studies using the present scale in other areas will increase its accuracy, making it more useful. However, to the best of our knowledge, this is the first study to develop a reliable and valid assessment scale focused on maternal daily lifestyle and psychology.

## Conclusion

The present study developed and examined the reliability and validity of a low birth weight risk assessment scale with items that reflect potential causes of low birth weight identified through a preliminary survey and previous research. The scale ultimately comprised the nine factors of “guidance at each checkup,” “adequate rest,” “support from husband,” “effects on fetus,” “support from society,” “support from family,” “effects of minor troubles,” “good lifestyle habits,” and “fall risk and lifestyle changes,” for which reliability (internal consistency) and construct validity (factor validity) were confirmed.

## Additional file

Additional file 1: For mothers with at least one preschool-aged child Survey on mothers’ living conditions during pregnancy. (DOCX 781 kb)

## Abbreviations

G–P analysis: Good–poor analysis
HSD test: Honestly significant difference test
Item–Total correlation: Correlation analysis between the item and scale
One-way ANOVA: One-way analysis of variance
SD: Standard deviation
Term: From 37 weeks gestation to less than 42 weeks gestation
