# Supplementary material for: A low-birth-weight risk assessment scale: development and validation through a questionnaire-based survey
Source: BMC Health Serv Res. 2019 Jan 18;19:48. doi: 10.1186/s12913-019-3886-7 (PMC6339392; doi:10.1186/s12913-019-3886-7)
Supplement: Supplementary file 1 — For mothers with at least one preschool-aged child Survey on mothers’ living conditions during pregnancy. (DOCX 781 kb) [file 12913_2019_3886_MOESM1_ESM.docx]

**For mothers with at least one preschool-aged child**

**Survey on mothers’ living conditions during pregnancy**

**Year 2015**

＊Please read these instructions before answering the questions in this questionnaire.

This questionnaire is directed at mothers with at least one preschool-aged child.

◎Please fill in the answers to the best of your knowledge and return the questionnaire directly to the researchers in the enclosed reply envelope.

◎Please note that your answers will be treated anonymously, and we will perform statistical analysis on the gathered data. Therefore, participating in this survey poses no risks to your privacy.

◎Also, you are not obligated to answer this questionnaire. You can even choose to stop at any time.

Please read the above statements and help us by answering these questions.

＊The questionnaire is divided into different sections. Some sections are meant to be answered only by mothers who did not have an employment during pregnancy or by mothers who were employed while pregnant. Thank you for following the directions, please proceed.

Guidelines for answering the questions:

■Please respond to this only if you are a mother with preschool-aged children.

■After filling in your answers into the questionnaire, please return it using the return envelope provided within one month after receipt of the questionnaire.

■ The basic information questions and the section A in this questionnaire are to be answered by all participants (pages 1–3), pages 4–8 are for mothers who did not work while pregnant [multiple choice questions in section B], and pages 9–15 are for mothers who worked while pregnant [multiple choice questions in section C]. You will need 15 to 20 minutes to answer the questionnaire. Please ensure that you fill in all sections that are applicable to your situation.

　＊If you have worked at least for a short while during any pregnancy, please fill the multiple choice questions in section C.

　　The following questions are for gathering basic information related to living conditions during pregnancy, including questions regarding your children and your past pregnancies. Please answer the questions related to your children (there is space for information for about two children). Please proceed by following these directions:

　　◆If you have only one preschool-aged child, answer only the column on part ① (regarding the first child).

　　◆If you have two preschool-aged children, answer both columns in part ① (regarding the first child) and in part ② (regarding the second child).

　　　◆If you have three preschool-aged children, we ask that you choose to answer the questions regarding the two children for whom your memory is stronger.

■For filling in the answers, please write a check mark ☑ on the provided square □, encircle the appropriate number to choose from a multiple choice question ○, and write down numbers and words within parentheses ( ).

■When filling in the questions regarding your preschool-aged children, please refer back to the maternal and child health notebooks, and try to remember your experience during pregnancy.

■First, please answer the following questions about yourself.

・please write a check mark ☑ on the provided square □, encircle the appropriate number to choose from a multiple choice question ○, and write down numbers and words within parentheses ( ).

・Please answer according to the guidelines present in “Q1” under the “Basic information column.”

　Basic information column: This question addresses all mothers. Please begin answering here.

common

Q1

　　　What is your current age? ➡ ( ) years

Q2

What is your height? ➡ ( ) cm

Q3

　　　What was your own birth weight? ➡ ( ) g

Q4

　　　Did you ever go on a diet prior to pregnancy?

➡□ Yes □ No

Q5

What is your current occupational status? (Please encircle only one choice 〇)

➡1. Full-time homemaker 2. full-time employee 3. self-employed

4. contract or part-time employee 5. Other ( )

Q6

　 Did you hear about childbirth from your parents/friends, etc., or from other sources?

If you answer “Yes,” please explain the details more specifically in your own words.

➡□ Yes

(please explain: )

□ No

Please continue here

　　　Q6-1 Is there a specific custom associated with childbirth that is practiced in your region?

If you check “Yes,” please explain the details in your own words.

➡□ Yes

(please explain )

□ No

Q7

What do you think the ideal birth weight (weight immediately after birth) of a baby should be? (Choose only one option ☑)

□ Less than 1500 g

□ Between 1500 and 2000 g

□ Between 2000 and 2300 g

□ Between 2300 and 2500 g

□ Between 2500 and 2800 g

□ Between 2800 and 3000 g

□ Between 3000 and 3500 g

□ 3500 g or more

1

| common  Q8  How many children do you have?  ➡ ( ) | | | | First child ( ) years old, second child ( ) years old,  third child ( ) years old, fourth child ( ) years old,  fifth child ( ) years old |
| --- | --- | --- | --- | --- |
| How many of your children are **preschool-aged** **(before elementary school)**?➡  **( )** | | | | |
|  | | | | |
| The section below **contains questions related to living conditions while you were pregnant with your child who is now a preschooler**.  Please refer to your child’s **Maternal and Child Health Handbook** to answer the following questions while recalling your pregnancy/ies. | | | | |
| ◆**If you have only one preschool-aged child, please answer only column ①.**  ◆If you have two preschool-aged children, please answer both columns ① (first child) and ② (second child).  ＊If you have three preschool-aged children, please answer the following questions based on the clearest memories of two of your three children. | | | | |
|  | | | | |
| What is the date of birth (month/day/year), sex, and birth weight of each of your preschool-aged children?  Q10 | | | | |
| **①** | (　) age | Preschool child  ( ) child | Birth weight ( ) g, Birth Weeks ( ) Days ( )  How old were you when this child was born? ➡ ( ) age  What was your pre-pregnancy body weight for this child?  ➡body weight ( ) kg  What was your maximum weight gain (kg) while you were pregnant with this child? ➡ ( ) kg increased. | |
|  | (Male / female)  ( years / months / days of life) | |  |  |
| **②** | ( ) age | Preschool child  ( ) child | Birth weight ( ) g, Birth week ( ) Week ( ) Day  How old were you when this child was born? ➡ ( ) age  What was your pre-pregnancy body weight for this child?  ➡body weight ( ) kg  What was your maximum weight gain (kg) while you were pregnant with this child? ➡ ( ) kg increased. | |
|  | (Male / female)  ( years / months / days life) | |  |  |

| 【multiple choice question A】This is to be answered by all mothers: The following items are related to your living conditions while you were pregnant with each child. |
| --- |
|  |
| About items 1–33, Column ① refers to (1^st^ child) · Column ② refers to (2^nd^ child) Please encircle ○ only the number that you think is most appropriate among the four options for each pregnancy. ( 4= Strongly Agree, 3 = Slightly Agree, 2 = Slightly Disagree 1 = Strongly Disagree) |

|  | Please provide answers for each child individually |  | **①** | | | |  | **②** | | | |
| --- | --- | --- | --- | --- | --- | --- | --- | --- | --- | --- | --- |
| № | I was pregnant with this child | | Strongly Agree: 4 | Slightly Agree: 3 | Slightly Disagree: 2 | Strongly Disagree: 1 |  | Strongly Agree: 4 | Slightly Agree:3 | Slightly Disagree: 2 | Strongly Disagree: 1 |
| 1 | I had severe morning sickness | | 4 | 3 | 2 | 1 |  | 4 | 3 | 2 | 1 |
| 2 | At one point, I believed that severe morning sickness was hereditary | | 4 | 3 | 2 | 1 |  | 4 | 3 | 2 | 1 |
| 3 | I experienced unpleasant symptoms, such as headaches | | 4 | 3 | 2 | 1 |  | 4 | 3 | 2 | 1 |
| 4 | During pregnancy checkups, I was given advice on my health status | | 4 | 3 | 2 | 1 |  | 4 | 3 | 2 | 1 |
| 5 | I consumed alcohol | | 4 | 3 | 2 | 1 |  | 4 | 3 | 2 | 1 |
| 6 | I thought about the effects of alcohol consumption on the fetus | | 4 | 3 | 2 | 1 |  | 4 | 3 | 2 | 1 |
| 7 | I thought about the effects of smoking on the fetus | | 4 | 3 | 2 | 1 |  | 4 | 3 | 2 | 1  common |
| 8 | I was always concerned about gaining weight　gain | | 4 | 3 | 2 | 1 |  | 4 | 3 | 2 | 1 |
| 9 | During pregnancy checkups, I was often given advice about my weight gain by medical staff | | 4 | 3 | 2 | 1 |  | 4 | 3 | 2 | 1 |
| 10 | I did not like going to checkups because the medical staff frequently advised me about my weight gain during previous pregnancy checkups | | 4 | 3 | 2 | 1 |  | 4 | 3 | 2 | 1 |
| 11 | I restricted my food intake as I was concerned about gaining weight | | 4 | 3 | 2 | 1 |  | 4 | 3 | 2 | 1 |
| 12 | I restricted my salt intake as I was concerned about my blood pressure | | 4 | 3 | 2 | 1 |  | 4 | 3 | 2 | 1 |
| 13 | I often experienced abdominal bloating | | 4 | 3 | 2 | 1 |  | 4 | 3 | 2 | 1 |
| 14 | I was able to lead an orderly lifestyle | | 4 | 3 | 2 | 1 |  | 4 | 3 | 2 | 1 |
| 15 | I tried to eat well-balanced meals | | 4 | 3 | 2 | 1 |  | 4 | 3 | 2 | 1 |
| 16 | I exercised adequately | | 4 | 3 | 2 | 1 |  | 4 | 3 | 2 | 1 |
| 17 | I slept well | | 4 | 3 | 2 | 1 |  | 4 | 3 | 2 | 1 |
| 18 | When I woke from my sleep, I felt refreshed | | 4 | 3 | 2 | 1 |  | 4 | 3 | 2 | 1 |
| 19 | I took holidays | | 4 | 3 | 2 | 1 |  | 4 | 3 | 2 | 1 |
| 20 | I took adequate rest | | 4 | 3 | 2 | 1 |  | 4 | 3 | 2 | 1 |
| 21 | At times I felt stressed | | 4 | 3 | 2 | 1 |  | 4 | 3 | 2 | 1 |
| 22 | I switched to shoes with low heels | | 4 | 3 | 2 | 1 |  | 4 | 3 | 2 | 1 |
| 23 | I was careful not to fall | | 4 | 3 | 2 | 1 |  | 4 | 3 | 2 | 1 |
| 24 | I refrained from driving a car | | 4 | 3 | 2 | 1 |  | 4 | 3 | 2 | 1 |
| 25 | There were some changes in my lifestyle compared to my pre-pregnancy days | | 4 | 3 | 2 | 1 |  | 4 | 3 | 2 | 1 |
| 26 | My husband thoroughly supported me | | 4 | 3 | 2 | 1 |  | 4 | 3 | 2 | 1 |
| 27 | My parents (including in-laws) thoroughly supported me | | 4 | 3 | 2 | 1 |  | 4 | 3 | 2 | 1 |
| 28 | People other than my husband and parents (including in-laws) thoroughly supported me | | 4 | 3 | 2 | 1 |  | 4 | 3 | 2 | 1 |
| 29 | I am satisfied with my husband’s support | | 4 | 3 | 2 | 1 |  | 4 | 3 | 2 | 1 |
| 30 | I am satisfied with my parents’ (including in-laws) support | | 4 | 3 | 2 | 1 |  | 4 | 3 | 2 | 1 |
| 31 | I am satisfied with the support that I received from others besides my husband and parents (including in-laws) | | 4 | 3 | 2 | 1 |  | 4 | 3 | 2 | 1 |
| 32 | I took advantage of the Maternity Mark (a symbol for expectant mothers) and experienced the effectiveness of the symbol in public settings | | 4 | 3 | 2 | 1 |  | 4 | 3 | 2 | 1 |
| 33 | I thought that the saying, “deliver them small and raise them big,” held true for me and my child(ren) | | 4 | 3 | 2 | 1 |  | 4 | 3 | 2 | 1 |

Q11

| ◆Did you experience fatigue while you were working when you were pregnant with your child(ren) who is/are now preschoolers?  (This question does not apply to you if you were a regular official, self-employed, a contractor/part-time employee) | |
| --- | --- |
| □ No →☑ If you checked “No” | **[multiple choice question B] - Please proceed to Q 11** |
|  |  |
| - Yes →☑ If you checked “Yes” | **[multiple choice question C] - Please proceed to Q 11** |

| [B vote] If you are not pregnant with any child, please fill this section. |
| --- |
| ☑ Please encircle ○ the number that you think is most appropriate, and fill the parentheses with your own words ( ). |

④

⑤

| [multiple choice questions]  **①**  Q12  Q12 | [multiple choice questions]  **②** |
| --- | --- |
| Multiple choice questions-№1 If you encircled “4. Strongly Agree” or “3. Slightly Agree” for the item “I had severe morning sickness,” please answer the following questions.  (*Otherwise, please proceed to Q13)  For women who did not have a job during pregnancy  ◆Duration of morning sickness:  Week ( ) to around Week ( ) of pregnancy  ◆During this period, I □ lost or □ gained ( ) kg  → □ lost □ gained ( ) kg (☑Check)  Please answer the following questions  Q12-1 Please mark all the morning sickness symptoms that you experienced with a check mark in the square □. Also, rank them from most severe to least severe, starting from 1, and write the number inside the parenthesis ( ).  □ (　) Vomited ( times per day)  □ (　) Odors made me sick.  □ (　) Felt sick on an empty stomach but felt better after eating.  □ (　) Unable to eat.  □ (　) Unable to eat or drink.  □ (　) Amount of saliva increased and made me uncomfortable.  □ (　) Taste of food changed  □ (　) Throat discomfort  □ (　) Experienced motion sickness more easily.  □ (　) Morning sickness only occurred in the morning.  □ (　) Other ( )  Q12-2 Please describe in detail the morning sickness symptoms when they were the worst.  ( )  Q12-3 What did you do to alleviate morning sickness symptoms? ( ) | Multiple choice questions-№1 If you encircled “4. Strongly Agree” or “3. Slightly Agree” for the item “I had severe morning sickness”, please answer the following questions.  (*Otherwise, please proceed to Q13)    ◆Duration of morning sickness:  Week ( ) to around Week ( ) of pregnancy  ◆During this period, I □ lost or □ gained ( ) kg  → □lost □gained ( ) kg (☑Check)  Please answer the following questions  　Q12-1 Please mark all the morning sickness symptoms that you experienced with a check mark in the square □. Also, rank them from most severe to least severe, starting from 1, and write the number inside the parenthesis ( ).  □ (　) Vomited ( times per day)  □ (　) Odors made me sick.  □ (　) Felt sick on an empty stomach but felt better after eating.  □ (　) Unable to eat.  □ (　) Unable to eat or drink.  □ (　) Amount of saliva increased and made me uncomfortable.  □ (　) Taste of food changed  □ (　) Throat discomfort  □ (　) Experienced motion sickness more easily.  □ (　) Morning sickness only occurred in the morning.  □ (　) Other ( )    Q12-2 Please describe, in detail, the morning sickness symptoms when they were the worst.  ( )  Q12-3 What did you do to alleviate morning sickness symptoms? ( ) |

5

| [multiple choice questions] Continuation of column ① | [multiple choice questions] Continuation of column ② |
| --- | --- |
| Q12-4 Was somebody present to support you while you were suffering from morning sickness?  (Multiple choice☑ )  □　No, I did not have anyone.  □　My husband supported me.  □　My parents and/or in-laws supported me.  □　My friends supported me.  □　My supervisor and colleagues at work supported me.  □　Other ( )  Q12-5 Were you ever hospitalized or treated for morning sickness? (＊Please answer to the best of your knowledge)  □I was never hospitalized or treated.  □I received outpatient treatment with complete bed rest at home.  □I received outpatient treatment with no restrictions on daily activities.  □I was hospitalized and received treatment.  Q13    Multiple choice question-№3 Please describe in detail the “symptoms of discomfort” during pregnancy. Please mark all your symptoms with a check mark in the square □. Also, rank them from most frequent to least frequent, starting from 1, and write the number inside the parenthesis ( ).  Q13  　□　I had no symptoms  □ (　) Increased drowsiness □ (　) Fever  □ (　) Headache □ (　) Constipation □ (　) Dizziness□ (　) Diarrhea □ (　) Emotional instability □ (　) Leg cramps　□ (　) Backache  □ (　) Varicose veins　□ (　) Other (  Q14  Multiple choice question-№4 If you encircled “4. Strongly Agree” or “3. Slightly Agree” for the item “During pregnancy checkups, I was given advice,” please answer the following questions. (*otherwise, please proceed to Q15).  Q14  Please encircle the numbers below that best describe the topics for which you received advice. (Multiple)  1. High blood pressure 2. Anemia 3. Urinary protein  4. Weight restriction 5. Edema 6. Bleeding 7. Suspected diabetes 8. Abnormal vaginal bleeding 9. Gingivitis 11. Dental cavities 10. Weight of the baby is not increasing 12. Bloating in the abdominal area 13. Pelvis size 14．Other ( )  If you encircled “12. Bloating in the abdominal area” in Q14, please answer the following questions.  Q14-1 Please tell us the measures you took against bloating in your abdominal area.  □ I did not do anything as it did not bother me much.  □ I persevered through the discomfort.  □ I sat down or laid down to rest.  □ Other ( )  If you encircled “13. Pelvis size” in Q14, please answer the following questions.  Q14-2 What did you hear about it specifically?  ( )  Next question. | Q12-4 Was somebody present to support you while you were suffering from morning sickness?  (Multiple choice☑ )  □　No, I did not have anyone.  □　My husband supported me.  □　My parents and/or in-laws supported me.  □　My friends supported me.  □　My supervisor and colleagues at work supported me.  □　Other ( )  For women who did not have a job during pregnancy      Q12-5 Were you ever hospitalized or treated for morning sickness? (＊＊Please answer to the best of your knowledge)  □I was never hospitalized or treated.  □I received outpatient treatment with complete bed rest at home.  □I received outpatient treatment with no restrictions on daily activities.  □I was hospitalized and received treatment.    Multiple choice question-№3 Please describe in detail the “symptoms of discomfort” during pregnancy. Please mark all your symptoms with a check mark in the square □. Also rank them from most frequent to least frequent, starting from 1, and write the number inside the parenthesis ( ).  　□　I had no symptoms  □ (　) Increased drowsiness□ (　) Fever  □ (　) Headache □ (　) Constipation □ (　) Dizziness□ (　) Diarrhea □ (　) Emotional instability  □ (　) Leg cramps □ (　) Backache  □ (　) Varicose veins □ (　) Other ( )  Multiple choice question-№4 If you encircled “4. Strongly Agree” or “3. Slightly Agree” for the item “During pregnancy checkups, I was given advice,” please answer the following questions. (*otherwise, please proceed to Q15).  Please encircle the numbers below that best describe the topics for which you received advice. (Multiple)  1. High blood pressure 2. Anemia 3. Urinary protein.  4. Weight restriction 5. Edema 6. Bleeding 7. Suspected diabetes 8. Abnormal vaginal bleeding 9. Gingivitis 11. Dental cavities 10. Weight of the baby is not increasing 12. Bloating in the abdominal area 13. Pelvis size 14．Other ( )  If you encircled “12. Bloating in the abdominal area” in Q14, please answer the following questions.  Q14-1 1 Please tell us the measures you took against bloating in your abdominal area.  □ I did not do anything as it did not bother me much.  □ I persevered through the discomfort.  □ I sat down or laid down to rest.  □ Other ( )  If you encircled “13. Pelvis size” in Q14, please answer the following questions.  Q14-2 What did you hear about it specifically? ( )  Next question. |
| [multiple choice questions] Continuation of column ① | [multiple choice questions] Continuation of column ② |
| Q14-3 Please describe to the best of your abilities what advice you were given at the pregnancy check-ups.  □I was never hospitalized or treated.  □I received outpatient treatment with complete bed rest at home.  □I received outpatient treatment with no restrictions on daily activities.  For women who did not have a job during pregnancy  □I was hospitalized and received treatment.  Q14-4 When you were pregnant with this child, were you given any advice about the health status of the fetus during your pregnancy check-ups?  □ Yes □ No (→Go to Q15)  If you answered “Yes” with a check mark, please answer the following question.  Q14-5 If you do not mind, please share what you learned.  ( )    Q15  Multiple choice question-№5 If you encircled “4. Strongly Agree” or “3. Slightly Agree” for the item “I consumed alcohol during pregnancy”, please answer the following questions. Please indicate the type of alcohol and amount of consumption.  Q15  □ Beer: ( ) bottles/cans per day, ( ) days per week  □ Others ( ): ( ) bottles/cans per day, ( ) days per week  Q16  Q16  Please tell us about your smoking status when you were pregnant with this child.  □ I never smoked  □ I stopped before becoming pregnant with this child.  □ I stopped once I learned I was pregnant with this child and have not smoked since. (Stopped smoking from Week ( ) of pregnancy)  □ I stopped for a while during the pregnancy of this child.  ➡(Stopped smoking between Week ( ) and Week ( ) of pregnancy)  □ I continued to smoke throughout the pregnancy of this child ➡(cigarettes per day)( )  Q17  Did your husband or any other person residing with you smoke while you were pregnant with this child? (Encircle only one option 〇)  １. They never smoked. (→Go to Q18)  ２. They stopped before I became pregnant with this child.  ３. They stopped once I learned I was pregnant with this child and have not smoked since. (Stopped smoking from Week ( ) of pregnancy)  ４. They stopped for a while during the pregnancy of this child.  ➡(Stopped smoking between Week ( ) and Week ( ) of pregnancy)  ５. They continued to smoke throughout the pregnancy of this child. ➡(cigarettes per day)( )  If you encircled “2,3,4, or 5” in Q17, please answer the following questions.  　 Q17-1 Where was the smoking area? (Multiple ☑ Yes)  　□ Same room as the expectant mother  □ Under the exhaust fan  □ Balcony | Q14-3 Please describe to the best of your abilities what advice you were given at the pregnancy check-ups.  □I was never hospitalized or treated.  □I received outpatient treatment with complete bed rest at home.  □I received outpatient treatment with no restrictions on daily activities.  □I was hospitalized and received treatment.  Q14-4 When you were pregnant with this child, were you given any advice about the health status of the fetus during your pregnancy check-ups?  □ Yes □ No (→Go to Q15)  If you answered “Yes” with a check mark, please answer the following question.  Q14-5 If you do not mind, please share what you learned.  ( )    Multiple choice question-№5 If you encircled “4. Strongly Agree” or “3. Slightly Agree” for the item “I consumed alcohol during pregnancy”, please answer the following questions. Please indicate the type of alcohol and amount of consumption.  □ Beer: ( ) bottles/cans per day, ( ) days per week  □ Others ( ): ( ) bottles/cans per day, ( ) days per week  Please tell us about your smoking status when you were pregnant with this child.  □ I never smoked  □ I stopped before becoming pregnant with this child.  □ I stopped once I learned I was pregnant with this child and have not smoked since. (Stopped smoking from Week ( ) of pregnancy)  □ I stopped for a while during the pregnancy of this child.  ➡(Stopped smoking between Week ( ) and Week ( ) of pregnancy)  □ I continued to smoke throughout the pregnancy of this child ➡(cigarettes per day)( )  Q17  Did your husband or any other person residing with you smoke while you were pregnant with this child? (Encircle only one option 〇)  １. They never smoked. (→Go to Q18)  ２. They stopped before I became pregnant with this child.  ３. They stopped once I learned I was pregnant with this child and have not smoked since. (Stopped smoking from Week ( ) of pregnancy)  ４. They stopped for a while during the pregnancy of this child.  ➡(Stopped smoking between Week ( ) and Week ( ) of pregnancy)  ５. They continued to smoke throughout the pregnancy of this child. ➡(cigarettes per day)( )  If you encircled “2,3,4, or 5” in Q17, please answer the following questions.  　 Q17-1 Where was the smoking area? (Multiple ☑ Yes)  　□ Same room as the expectant mother  □ Under the exhaust fan  □ Balcony |
| [multiple choice questions] Continuation of column ① | [multiple choice questions]Continuation of column ② |
| □ Outside the front door of home  □ Smoked in places other than home  Q18  Q18  Multiple choice question-№8 If you encircled “4. Strongly Agree” or “3. Slightly Agree” for the item “I was concerned about my weight,” during pregnancy, please answer the following questions. Please mark all the reasons for the concern about your weight gain with a check mark in the square □. Also, rank the options from most concerning to least concerning, starting from 1, and write the number inside the parenthesis ( ).  □ (　) Because I was given advice at the hospital.  □ (　) Because I was concerned about my figure.  □ (　) Because I was told by others around me.  □ (　) Because I read information in magazines.  □ (　) Because I read information on the Internet.  □ (　) Others  Q18-1 What were some words or information that made an impression on you? ( )  Q19  Q19  Please tell us about your housing situation and whether stairs were present or not while you were pregnant with this child.  □ Detached house ➡ [□with /□without stairs]  □ Condominium / apartment  ➡ [( )^th^ floor,□ with /□ without elevator]   - Others ( )   Q20  Q20  Multiple choice question-№21 If you encircled “4. Strongly Agree” or “3. Slightly Agree” for the item “At times I felt stressed,” please answer the following questions. (*Otherwise, please proceed to Q21). Please tell us about the cause of the stress. ( )  Q21  Q21  Multiple choice question-№25 If you encircled “4. Strongly Agree” or “3. Slightly Agree” for the item “There were some changes in my lifestyle”, please answer the following questions.  (* Otherwise, please proceed to Q22) What specific changes did you make? ( )  Q22    What was the most difficult thing you had to endure while you were pregnant with this child? (Choose only one ☑)  □ Nothing in particular  □ Morning sickness　□ Bloating in the abdominal area  □ Edema of legs　□ Mood swings  □ Others ( )  Q23  Did you attend maternity or birthing classes while you were pregnant with this child?  　 □Yes □No  Q24  Q24  What was the reason you decided to go to the hospital for before giving birth to this child?  □　Natural birth　　□　Induced labor  □　Cesarean section  □　Others ( )  Q25  Q25  Please tell us about the delivery of this child.  　　□ Natural birth □ Induced labor  □ Cesarean section　□Others ( )  Q26  What was the employment status of the baby’s father while you were pregnant with this child?    [multiple choice questions] Continuation of column ① | □ Outside the front door of home  □ Smoked in places other than home  Multiple choice question-№8 If you encircled “4. Strongly Agree” or “3. Slightly Agree” for the item “I was concerned about my weight,” during pregnancy, please answer the following questions. Please mark all the reasons for the concern about your weight gain with a check mark in the square □. Also, rank the options from most concerning to least concerning, starting from 1, and write the number inside the parenthesis ( ).  □ (　) Because I was given advice at the hospital.  □ (　) Because I was concerned about my figure.  □ (　) Because I was told by others around me.  □ (　) Because I read information in magazines.  □ (　) Because I read information on the Internet.  □ (　) Others  Q18-1 What were some words or information that made an impression on you? ( )  Please tell us about your housing situation and whether stairs were present or not present while you were pregnant with this child.  F For women who did not have a job during pregnancy  □ Detached house ➡ [□with /□without stairs]  □ Condominium / apartment  ➡ [( )^th^ floor,□ with /□ without elevator]   - Others ( )   Multiple choice question-№21 If you encircled “4. Strongly Agree” or “3. Slightly Agree” for the item “At times I felt stressed”, please answer the following questions. (*Otherwise, please proceed to Q21). Please tell us about the cause of the stress. ( )  Multiple choice question-№25 If you encircled “4. Strongly Agree” or “3. Slightly Agree” for the item “There were some changes in my lifestyle”, please answer the following questions.  (* Otherwise please proceed to Q22). What specific changes did you make? ( )  Q22    What was the most difficult thing you had to endure while you were pregnant with this child? (Choose only one ☑)  □Nothing in particular  □Morning sickness　□Bloating in the abdominal area  □Edema of legs　□Mood swings  □ Others ( )  Q23  Did you attend maternity or birthing classes while you were pregnant with this child?  □Yes □  What was the reason you decided to go to the hospital for before giving birth to this child?  □　Natural birth　　□　Induced labor  □　Cesarean section  □　Others ( )  Please tell us about the delivery of this child.  　　□ Natural birth. □ Induced labor  Q26  　 □ Cesarean section　□　Other  What was the employment status of the baby’s father while you were pregnant with this child?  (Encircle only one number 〇)  [multiple choice questions] Continuation of column ② |
| (Encircle only one number 〇)  ➡1. Full-time employee　2. Contractor or part-time employee　3. Self-employed  4. Unemployed 5. Others ( )  Q27  Q27  Did your husband take childcare leave for this child? (This does not include holidays or paid vacation)  □ Yes □ No  For women who did not have a job during pregnancy  Q28  Q28    Did your husband take spousal maternity leave for this child?  (This does not include holidays or paid vacation)    □ Yes □ No  Q29  Was your husband on a shorter working-hour system for this child?  □ Yes □ No  Q30  Q30    Was your husband on a nursing care leave system for this child? (This does not include holidays or paid vacation)  □ Yes □ No  Q31  Multiple choice question-№32 If you encircled “4. Strongly Agree” or “3. Slightly Agree” for the item about looking for the Maternity Mark while you were pregnant with this child, please answer the following questions. Please describe how you used it concretely. ( ) | (Encircle only one number 〇)  ➡1. Full-time employee　2. Contractor or part-time employee　3. Self-employed  4. Unemployed 5. Others ( )  Did your husband take childcare leave or this child? (This does not include holidays or paid vacation)  □ Yes □ No    Did your husband take spousal maternity leave for this child?  (This does not include holidays or paid vacation)  □ Yes □ No  Q29  Was your husband on a shorter working-hour system for this child?  □ Yes □ No    Was your husband on a nursing care leave system for this child? (This does not include holidays or paid vacation)  □ Yes □ No  Q31  Multiple choice question-№32 If you encircled “4. Strongly Agree” or “3. Slightly Agree” for the item about using the Maternity Mark while you were pregnant with this child, please answer the following questions. Please describe how you used it concretely. ( ) |

Q26

◇ End of the questionnaire for women who did not have a job during pregnancy.

Thank you for your cooperation.

| [multiple choice question C] The following section contains questions related to working conditions of the expectant mother. (This question does not apply to you if you were a regular official, self-employed, a contractor/part-time employee)  For women who worked while pregnant |
| --- |
| ☑ Please encircle ○ the number that you think is the most appropriate and write within the parenthesis ( ). |

| [multiple choice question C]  **①**  Q12 | [multiple choice question C]  **②** |
| --- | --- |
| Multiple choice question-№1 If you encircled “4. Strongly Agree” or “3. Slightly Agree” for the item “I had severe morning sickness,” please answer the following questions.  (*Otherwise, please proceed to Q13)  ◆Duration of morning sickness:  Week ( ) to around Week ( ) of pregnancy  ◆During this period, I □ lost or □ gained ( ) kg  → □lost o　□gained ( ) kg (☑Check)  Q12-1 Please mark all the morning sickness symptoms that you experienced with a check mark in the square □. Also, rank them from most severe to least severe, starting from 1, and write the number inside the parenthesis ( ).  □ (　) Vomited ( times per day)  □ (　) Odors made me sick.  □ (　) Felt sick on an empty stomach but felt better after eating.  □ (　) Unable to eat.  □ (　) Unable to eat or drink.  □ (　) Amount of saliva increased and made me uncomfortable.  □ (　) Taste of food changed  □ (　) Throat discomfort.  □ (　) Experienced motion sickness more easily.  □ (　) Morning sickness only occurred in the morning.  □ (　) Others (　　　　　　　　　　　)    Q12-5 Were you ever hospitalized or treated for morning sickness? (＊Please answer to the best of your knowledge)  □ I was never hospitalized or treated.  □ I received outpatient treatment with complete bed rest at home.  □ I received outpatient treatment with no restrictions on daily activities.  □ I was hospitalized and received treatment.  Q13    Multiple choice question-№3 Please describe in detail the “symptoms of discomfort” during pregnancy. Please mark all your symptoms with a check mark in the square □. Also, rank them from most frequent to the least frequent, starting from 1, and write the number inside the parenthesis ( ).  Q13  　□　I had no symptoms  □ (　) Increased drowsiness　　　□ (　) Fever  □ (　) Headache　□ (　) Constipation　□ (　) Dizziness□ (　) Diarrhea　　□ (　) Emotional instability | Multiple choice question-№1 If you encircled “4. Strongly Agree” or “3. Slightly Agree” for the item “I had severe morning sickness,” please answer the following questions.  Q12  (*Otherwise, please proceed to Q13．)  ◆Duration of morning sickness:  Week ( ) to around Week ( ) of pregnancy  ◆During this period, I □ lost or □ gained ( ) kg  → □lost o　□gained ( ) kg (☑Check)  Q12-1 Please mark all the morning sickness symptoms that you experienced with a check mark in the square □. Also, rank them from most severe to least severe, starting from 1, and write the number inside the parenthesis ( ).  □ (　) Vomited ( times per day)  □ (　) Odors made me sick.  □ (　) Felt sick on an empty stomach but felt better after eating.  □ (　) Unable to eat.  □ (　) Unable to eat or drink.  □ (　) Amount of saliva increased and made me uncomfortable.  □ (　) Taste of food changed  □ (　)Throat discomfort.  □ (　) Experienced motion sickness more easily.  □ (　) Morning sickness only occurred in the morning.  □ (　) Others (　　　　　　　　　　　)    Q12-5 Were you ever hospitalized or treated for morning sickness? (＊Please answer to the best of your knowledge)  □ I was never hospitalized or treated.  □ I received outpatient treatment with complete bed rest at home.  □ I received outpatient treatment with no restrictions on daily activities.  □ I was hospitalized and received treatment.    Multiple choice question-№3 Please describe in detail the “symptoms of discomfort” during pregnancy. Please mark all your symptoms with a check mark in the square □. Also, rank them from most frequent to the least frequent, starting from 1, and write the number inside the parenthesis ( ).  　□　I had no symptoms  □ (　) Increased drowsiness　　　□ (　) Fever  □ (　) Headache　□ (　) Constipation　□ (　) Dizziness□ (　) Diarrhea　　□ (　) Emotional instability |
| [multiple choice question C] Continuation of column ① | [multiple choice question C] Continuation of column ② |
| For women who worked while pregnant  　 □ (　) Leg cramps　□ (　) Backache  □ (　) Varicose veins　□ (　) Others (　　　　)  Q14  Q14  Multiple choice question-№4 If you encircled “4. Strongly Agree” or “3. Slightly Agree” for the item “During pregnancy checkups, I was given advice,” please answer the following questions.  (*otherwise, please proceed to Q15).  Please encircle the numbers below that best describe the topics for which you received advice. (Multiple)  1. High blood pressure　2. Anemia　3. Urinary protein  4. Weight restriction　　　5. Edema　　　6. Bleeding 7. Suspected diabetes　8. Abnormal vaginal bleeding　　9. Gingivitis 11. Dental cavities　 10. Weight of the baby is not increasing　12. Bloating in the abdominal area  13. Pelvis size 14.Others (　　　　　　　　　　　)  If you encircled “12. Bloating in the abdominal area” in Q14, please answer the following question.  Q14-1 Please describe what measures you took when you experienced bloating in your abdominal area.  □ I did not do anything as it did not bother me much.  □ I persevered through the discomfort.  □ I sat down or laid down to rest.  □ Others (　　　　　　　　　　　　　　　　)  If you encircled “13. Pelvis size” in Q14, please answer the following questions.  Q14-2 Please share what you learned  (　　　　　　　　　　　　　　　　　　　　　)  Q14-3 Please describe to the best of your abilities what advice were you given at the pregnancy check-ups.  □ I was never hospitalized or treated.  □ I received outpatient treatment with complete bed rest at home.  □ I received outpatient treatment with no restrictions on daily activities.  □ I was hospitalized and received treatment.  Q14-4 When you were pregnant with this child, were you given any advice about the health status of the fetus during your pregnancy check-ups?  □ Yes　　　　　□ No (→Go to Q15)  If you marked “Yes” with a check mark, please answer the following questions.  Q14-5 If you do not mind, please share what you learned.  (　　　　　　　　　　　　　　　 　)    Q15  Multiple choice question-№5 If you encircled “4. Strongly Agree” or “3. Slightly Agree” for the item “I consumed alcohol during pregnancy,” please answer the following questions. Please indicate the type of alcohol and amount of consumption.  □ Beer: ( ) bottles/cans per day, ( ) days per week  □ Others ( ): ( ) bottles/cans per day, ( ) days per week | □ (　) Leg cramps　□ (　) Backache  □ (　) Varicose veins　□ (　) Others (　　　　)  Multiple choice question-№4 If you encircled “4. Strongly Agree” or “3. Slightly Agree” for the item “During pregnancy checkups, I was given advice,” please answer the following questions.  (*otherwise, please proceed to Q15).  Please encircle the numbers below that best describe the topics for which you received advice. (Multiple)  1. High blood pressure　2. Anemia　3. Urinary protein  4. Weight restriction　　　5. Edema　　　6. Bleeding 7. Suspected diabetes　8. Abnormal vaginal bleeding　　9. Gingivitis 11. Dental cavities　 10. Weight of the baby is not increasing　12. Bloating in the abdominal area  13. Pelvis size 14.Others (　　　　　　　　　　　)  If you encircled “12. Bloating in the abdominal area” in Q14, please answer the following question.  Q14-1 Please describe what measures you took when you experienced bloating in your abdominal area.  □ I did not do anything as it did not bother me much.  □ I persevered through the discomfort.  □ I sat down or laid down to rest.  □ Others (　　　　　　　　　　　　　　　　)  If you encircled “13. Pelvis size” in Q14, please answer the following questions.  Q14-2 Please share what you learned  (　　　　　　　　　　　　　　　　　　　　　)  .  Q14-3 Please describe to the best of your abilities what advice were you given at the pregnancy check-ups.  □ I was never hospitalized or treated.  □ I received outpatient treatment with complete bed rest at home.  □ I received outpatient treatment with no restrictions on daily activities.  □ I was hospitalized and received treatment.  Q14-4 When you were pregnant with this child, were you given any advice about the health status of the fetus during your pregnancy check-ups?  □ Yes　　　　　□ No (→Go to Q15)  If you marked “Yes” with a check mark, please answer the following questions.  Q14-5 If you do not mind, please share what you learned.  (　　　　　　　　　　　　　　　 　)  Q15    Multiple choice question-№5 If you encircled “4. Strongly Agree” or “3. Slightly Agree” for the item “I consumed alcohol during pregnancy,” please answer the following questions. Please indicate the type of alcohol and amount of consumption.  □ Beer: ( ) bottles/cans per day, ( ) days per week  □ Others ( ): ( ) bottles/cans per day, ( ) days per week |
| [multiple choice question C] Continuation of column ①  Q16 | [multiple choice question C] Continuation of column ② |
| Please tell us about your smoking status when you were pregnant with this child.  Q16  □ I never smoked  □ I stopped before becoming pregnant with this child.  □ I stopped once I learned I was pregnant with this child and have not smoked since. (Stopped smoking from Week ( ) of pregnancy)  □ I stopped for a while during the pregnancy of this child.  ➡(Stopped smoking between Week ( ) and Week ( ) of pregnancy)  □ I continued to smoke throughout the pregnancy of this child　➡(cigarettes per day)(　　)  Q17  Did your husband or any other person residing with you smoke while you were pregnant with this child? (Only one〇)  １. They never smoked. (→To Q18)  ２. They stopped before I became pregnant with this child.  ３.They stopped once I learned I was pregnant with this child and have not smoked since. (Stopped smoking from Week ( ) of pregnancy)  ４. They stopped for a while during the pregnancy of this child.  ➡(Stopped smoking between Week ( ) and Week ( ) of pregnancy)  ５. They continued to smoke throughout the pregnancy of this child. ➡(cigarettes per day)(　　)  If you encircled “2,3,4, or 5” in Q17, please answer the following questions.  　 Q17-1 Where was the smoking area? (Multiple ☑ Yes)  　□ Same room as the expectant mother  □ Under the exhaust fan  □ Balcony  □ Outside the front door of home  □ Smoked in places other than home  Q18  Multiple choice question-№8 If you encircled “4. Strongly Agree” or “3. Slightly Agree” for the item “I was concerned about my weight” during pregnancy, please answer the following questions. Please mark all the reasons for your concern about your weight gain with a check mark in the square □. Also, rank them from most concerning to least concerning, starting from 1, and write the number inside the parenthesis ( ).  □ (　) Because I was given advice at the hospital.  □ (　) Because I was concerned about my figure.  □ (　) Because I was told by others around me.  □ (　) Because I read information in magazines.  □ (　) Because I read information on the Internet.  □ (　) Others    Q18-1 What were some words or information that made an impression on you? (　　　　　　　　　　　　)  Q19  Q19  Please tell us about your housing situation and whether stairs were present or not while you were pregnant with this child.  □　Detached house　➡ [□ with/□ without stairs]  □　Condominium / apartment  ➡ [( )^th^ floor, □ with/□ without elevator]   - Others (　　　　　　　　　　　)   Q21  Q21 | Please tell us about your smoking status when you were pregnant with this child.  □ I never smoked  □ I stopped before becoming pregnant with this child.  □ I stopped once I learned I was pregnant with this child and have not smoked since. (Stopped smoking from Week ( ) of pregnancy)  For women who worked while pregnant  □ I stopped for a while during the pregnancy of this child.  ➡(Stopped smoking between Week ( ) and Week ( ) of pregnancy)  □ I continued to smoke throughout the pregnancy of this child　➡(cigarettes per day)(　　)  Q17  Did your husband or any other person residing with you smoke while you were pregnant with this child? (Only one〇)  １. They never smoked. (→To Q18)  ２. They stopped before I became pregnant with this child.  ３.They stopped once I learned I was pregnant with this child and have not smoked since. (Stopped smoking from Week ( ) of pregnancy)  ４. They stopped for a while during the pregnancy of this child.  ➡(Stopped smoking between Week ( ) and Week ( ) of pregnancy)  ５. They continued to smoke throughout the pregnancy of this child. ➡(cigarettes per day)(　　)  If you encircled “2,3,4, or 5” in Q17, please answer the following questions.  　 Q17-1 Where was the smoking area? (Multiple ☑ Yes)  　□ Same room as the expectant mother  □ Under the exhaust fan  □ Balcony  □ Outside the front door of home  □ Smoked in places other than home  Q18  Multiple choice question-№8 If you encircled “4. Strongly Agree” or “3. Slightly Agree” for the item “I was concerned about my weight” during pregnancy, please answer the following questions. Please mark all the reasons for your concern about your weight gain with a check mark in the square □. Also, rank them from most concerning to least concerning, starting from 1, and write the number inside the parenthesis ( ).  □ (　) Because I was given advice at the hospital.  □ (　) Because I was concerned about my figure.  □ (　) Because I was told by others around me.  □ (　) Because I read information in magazines.  □ (　) Because I read information on the Internet.  □ (　) Others    Q18-1 What were some words or information that made an impression on you? (　　　　　　　　　　　　)  Please tell us about your housing situation and whether stairs were present or not present while you were pregnant with this child.  □　Detached house　➡ [□ with/□ without stairs]  □　Condominium / apartment  ➡ [( )^th^ floor, □with/□ without elevator]   - Others (　　　　　　　　　　　)   Q21  Q21 |
| [multiple choice question C] Continuation of column ① | [multiple choice question C] Continuation of column ② |
| Q20  Multiple choice question-№21 If you encircled “4. Strongly Agree” or “3. Slightly Agree” for the item “At times I felt stressed,” please answer the following questions. (*Otherwise, please proceed to Q21). Please describe the cause of the stress.  For women who worked while pregnant  　( 　)  Q21  Multiple choice question-№25 If you encircled “4. Strongly Agree” or “3. Slightly Agree” for the item “There were some changes in my lifestyle,” while you were pregnant with this child, please answer the following questions.  (* Otherwise, please proceed to Q22). What specific changes did you make? (　　　　　　　　　　　)  Q22    What was the most difficult thing you had to endure while you were pregnant with this child? (Only one ☑)  □ Nothing in particular  □ Morning sickness　□ Bloating in the abdominal area  □ Edema of legs　□ Mood swings  □ Others (　　　　　　　　　　　　　　　　　)  Q23　３    Did you attend maternity or birthing classes while you were pregnant with this child?  　 □ Yes　　　　□ No  Q24  What was the reason you decided to go to the hospital for before giving birth to this child?  □　Natural birth　　□　Induced labor  □　Cesarean section  □　Others (　　　　　　　　　　　　　)  Q25  Please describe the delivery of this child.  　 □ Natural birth □ Induced labor  □ Cesarean section　□ Others (　　　　　)  Q26  What was the employment status of the baby’s father while you were pregnant with this child?  (Encircle only one number 〇)  Q23  ➡1. Full-time employee  　2. Contract or part-time employee  3. Self-employed  4. Unemployed  5. Other (　　　　　　　)    Q27  Did your husband take parental work leave for this child? (This does not include holidays or paid vacation).  □ Yes　　□ No  Q28  Q28    Did your husband take spousal maternity work leave for this child?  (This does not include holidays or paid vacation).  □ Yes　　□ No  Q29  Q29  Was your husband on a shorter working-hour system for this child?  □ Yes　　□ No  12    【multiple choice question C】Continuation of column ① | Q20  Multiple choice question-№21 If you encircled “4. Strongly Agree” or “3. Slightly Agree” for the item “At times I felt stressed,” please answer the following questions. (*Otherwise, please proceed to Q21). Please describe the cause of the stress.  　( 　)  Q21  Multiple choice question-№25 If you encircled “4. Strongly Agree” or “3. Slightly Agree” for the item “There were some changes in my lifestyle,” while you were pregnant with this child, please answer the following questions.  (* Otherwise, please proceed to Q22). What specific changes did you make? (　　　　　　　　　　　)  Q22    What was the most difficult thing you had to endure while you were pregnant with this child? (Only one ☑)  □ Nothing in particular  □ Morning sickness　□ Bloating in the abdominal area  □ Edema of legs　□ Mood swings  □ Others (　　　　　　　　　　　　　　　　　)  Q23    Did you attend maternity or birthing classes while you were pregnant with this child?  　 □ Yes　　　　□ No  Q24  What was the reason you decided to go to the hospital for before giving birth to this child?  □　Natural birth　　□　Induced labor  □　Cesarean section  □　Others (　　　　　　　　　　　　　)  Q25  Please describe the delivery of this child.  　 □ Natural birth □ Induced labor  □ Cesarean section　□ Others (　　　　　)  Q26  What was the employment status of the baby’s father while you were pregnant with this child?  (Encircle only one number〇)  Q23  ➡1. Full-time employee  　2. Contract or part-time employee  3. Self-employed  4. Unemployed  5. Other (　　　　　　　)  Q27    Did your husband take parental work leave for this child? (This does not include holidays or paid vacation).  □ Yes　　□ No  Did your husband take spousal maternity work leave for this child?  (This does not include holidays or paid vacation).  □ Yes　　□ No  Was your husband on a shorter working-hour system for this child?  □ Yes □ No    【multiple choice question C】Continuation of column ② |
| Q30    Was your husband on a nursing care leave for this child? (This does not include holidays or paid vacation).  □ Yes　　　　　□ No  Q31  Multiple choice question-№32 If you encircled “4. Strongly Agree” or “3. Slightly Agree” for the item about using the Maternity Mark while you were pregnant with this child, please answer the following questions. Please describe in detail. (　　　　　　　　　　　　　　　　　　　　　) | Q30  Was your husband on a nursing care leave for this child? (This does not include holidays or paid vacation).  □ Yes　　　　　□ No  Q31    Multiple choice question-№32 If you encircled “4. Strongly Agree” or “3. Slightly Agree” for the item about using the Maternity Mark while you were pregnant with this child, please answer the following questions. Please describe in detail. (　　　　　　　　　　　　　　　　　　　　　) |

For women who worked while pregnant

**The following section contains questions related to working conditions for the expectant mother.**

|  | **[multiple choice questions C: For women who worked while pregnant]**  Please answer for each individual pregnancy |  | ① | | | |  | ② | | | |
| --- | --- | --- | --- | --- | --- | --- | --- | --- | --- | --- | --- |
| № | I was pregnant with this child | | Strongly Agree:4 | Slightly Agree: 3 | Slightly Disagree: 2 | Strongly Disagree: 1 |  | Strongly Agree: 4 | Slightly Agree:3 | Slightly Disagree: 2 | Strongly Disagree: 1 |
| 34 | I often worked overtime during my first trimester (Week 4 to week 15) | | 4 | 3 | 2 | 1 |  | 4 | 3 | 2 | 1 |
| 35 | I often worked overtime during my second trimester (Week 16 to week 27) | | 4 | 3 | 2 | 1 |  | 4 | 3 | 2 | 1 |
| 36 | I often worked overtime during my third trimester (Week 28 to week 39) | | 4 | 3 | 2 | 1 |  | 4 | 3 | 2 | 1 |
| 37 | I considered people at work to be very considerate. | | 4 | 3 | 2 | 1 |  | 4 | 3 | 2 | 1 |
| 38 | I took time off from work because I did not feel well. | | 4 | 3 | 2 | 1 |  | 4 | 3 | 2 | 1 |
| 39 | I considered quitting my job. | | 4 | 3 | 2 | 1 |  | 4 | 3 | 2 | 1 |

| For women who worked while pregnant (Please answer the question below). |
| --- |

Q32

For women who worked while pregnant

| [multiple choice question] Continuation of column ① | [multiple choice question C] Continuation of column ②  Q32 |
| --- | --- |
| Please tell us about your work arrangement during pregnancy. (Encircle only one 〇)  １ Full-time homemaker 　２ Full-time employee  ３ Part-time (temporary, contract employee)  ４ Self-employment  ５ Other ( )  * If you encircled “1. Full-time homemaker,” this is the end of the questions related to the pregnancy of your preschool child.  【multiple choice question C】Please proceed to Q32 in the column ②.  If you encircled “2. Full-time employee, 3. Part-time and four. Self-employed,” please answer the questions below.  Q32-1 Please give us details about your occupation. (Encircle only one ☑)  　　□ Administrative clerk　□ Teacher　□ Hair stylist  □ Cashier clerk □ Janitor □ Sales clerk  □ Waitress □ Restaurant business　□ Person handing out fliers　□Taxi driver □ Shipping/delivery business 　□ Nursing care staff　□ Nurse  □ pharmacist □ Physician/Dentist  □ Physiotherapist/occupational therapist  □ Dental hygienist　□ Other medical staff  □ Other ( )  Q32-2 Please describe the working period during pregnancy. (Please mark only one answer with a check mark in the square □).  □ Before learning about the pregnancy to week ( )  □ Week ( ) to Week ( ) of pregnancy  Please answer the following questions about your posture in the work environment.  Q32-3 Which position best describes your predominant working posture? (Please mark only one answer with a check mark in the square □).  □ Standing □ Half-crouching □ Sitting  □ Others ( )  Q32-4 How long were you in this position (hours/day)?  (Please mark only one answer with a check mark in the☑ )  □1–2 hours □ 3–4 hours □5–6 hours  □ 7–8 hours □ 8 hours or more  Next question.  Q32-5 During pregnancy, did you work at a job with night shifts? If so, please mention the number of night shifts you worked.  □No (→ To Q33) □ Once a week □ 2–3 times a week □ 4–5 times a week  □ Once a month  Please answer this question if you worked “night shifts.”  Q32-6 How many weeks into your pregnancy were you working night shifts?  ➡ ( ) I worked night shifts until week ( ). | Please tell us about your work arrangement during pregnancy. (Encircle only one 〇)  １ Full-time homemaker 　２ Full-time employee  ３ Part-time (temporary, contract employee)  ４ Self-employment  ５ Other ( )  * If you encircled “1. Full-time homemaker,” this is the end of the questions related to the pregnancy of your preschool child.  【multiple choice question C】Please proceed to Q32 in the column ②.  If you encircled “2. Full-time employee, 3. Part-time and 4. Self-employed,” please answer the questions below.  Q32-1 Please give us details about your occupation. (Encircle only one ☑)  　　□ Administrative clerk　□ Teacher　□ Hair stylist  □ Cashier clerk □ Janitor □ Sales clerk  □ Waitress □Restaurant business  □Person handing out fliers　□Taxi driver  □ Shipping/delivery business 　□ Nursing care staff　□ Nurse　□ pharmacist  □ Physician/Dentist  □ Physiotherapist/occupational therapist  □ Dental hygienist　□ Other medical staff  □ Other ( )  Q32-2 Please describe the working period during pregnancy. (Please mark only one answer with a check mark in the square □).  □ Before learning about the pregnancy to week ( )  □ Week ( ) to Week ( ) of pregnancy  Please answer the following questions about your posture in the work environment.  Q32-3 Which position best describes your predominant working posture? (Please mark only one answer with a check mark in the square □).  □ Standing □ Half-crouching □ Sitting.  □ Others ( )  Q32-4 How long were you in this position (hours/day)?  (Please mark only one answer with a check mark in the☑ )  □1–2 hours □3–4 hours □5–6 hours  □ 7–8 hours □8 hours or more  Next question.  Q32-5 During pregnancy, did you work at a job with night shifts? If so, please mention the number of night shifts you worked.  □No (→ To Q33) □ Once a week □ 2–3 times a week □ 4–5 times a week  □ Once a month  Please answer this question if you worked “night shifts.”  Q32-6 How many weeks into your pregnancy were you working night shifts?  ➡ ( ) I worked night shifts until week ( ). |
| [multiple choice question C] Continuation of column ① | [multiple choice question C] Continuation of column ② |
| Multiple choice question-№34~36 If you encircled “4. Strongly Agree” or “3. Slightly Agree” for the item “I often worked overtime during pregnancy,”,, please answer the following questions. Please describe in detail the frequency of “overtime work.”  (*Otherwise, please go to Q34)  □ Once a week □2–3 times a week  □4–5 times a week.□ Once a month  Q34  What was your main mode of transportation to work during pregnancy?  (Please mark only one answer, the most frequent mode, with a check mark in the square □)  □ Walking □ Cycling □ Bus □ Municipal railway  □ Japan Railway (JR) □ Private car  □ Driven by a member of the household  Q34-1 How much time did you spend on your daily commute?  □ Within 30 minutes □ 30 minutes to 1 hour □ 1 hour to 90 minutes □ 90 minutes to 2 hours  □ 2 hours or more  Q35  Multiple choice question-№37 If you encircled “4. Strongly Agree” or “3. Slightly Agree” for the item “I considered people at work to be very considerate,” please answer the following question. (*Otherwise, please go to Q36).  What kind of special care (consideration) was offered? Please describe below.  ( )  Q36  Multiple choice question-№39 If you encircled “4. Strongly Agree” or “3. Slightly Agree” for the item “I considered quitting my job,” please answer the following question. **(***Others please go to **Q37)**  Please tell us the reason why. ( )    Q37  Did you take childcare work leave for the birth of this child?  □ Yes □ No→ ( to **Q37-2)**  Q37-1 Did you return to work after your childcare work leave?  □ I went back to my original work place.  　□ I quit my previous job and found another one.  □ I did not go back to the workplace.    Q37-2 Were you on a shorter working-hour system for this child?  □ Yes □ No  Q37-3 Were you on a nursing care work leave for this child?  □ Yes □ No | Multiple choice question-№34~36 If you encircled “4. Strongly Agree” or “3. Slightly Agree” for the item “I often worked overtime during pregnancy,” please answer the following questions. Please describe in detail the frequency of “overtime work.”  (* Otherwise, please go to Q34)  □ Once a week □2–3 times a week  □4–5 times a week □ Once a month  Q34  What was your main mode of transportation to work during pregnancy?  (Please mark only one answer, the most frequent mode, with a check mark in the square □)  □ Walking □ Cycling □ Bus □ Municipal railway  □ Japan Railway (JR) □ Private car  □ Driven by a member of the household  Q34-1 How much time did you spend on your daily commute?  □ Within 30 minutes □ 30 minutes to 1 hour □ 1 hour to 90 minutes □ 90 minutes to 2 hours  □ 2 hours or more  Q35  Multiple choice question-№37 If you encircled “4. Strongly Agree” or “3. Slightly Agree” for the item “I considered people at work to be very considerate,” please answer the following question. (*Otherwise, please go to Q36).  What kind of special care (consideration) was offered? Please describe below.  ( )  Q36  Multiple choice question-№39 If you encircled “4. Strongly Agree” or “3. Slightly Agree” for the item “I considered quitting my job,” please answer the following question. **(***Others please go to **Q37)**  Please tell us the reason why. ( )    Q37  Did you take childcare work leave for the birth of this child?  □ Yes □ No→ ( to **Q37-2)**  Q37-1 Did you return to work after your childcare work leave?  □ I went back to my original work place.  　□ I quit my previous job and found another one.  □ I did not go back to the workplace.    Q37-2 Were you on a shorter working-hour system for this child?  □ Yes □ No  Q37-3 Were you on a nursing care work leave for this child?  □ Yes □ No |

End of the questionnaire for women who worked during pregnancy. Thank you for your cooperation.

15
